# Supplementary material for: Taxon-dependent diversity response along a temperate elevation gradient covered by grassland
Source: PeerJ. 2024 Jun 21;12:e17375. doi: 10.7717/peerj.17375 (PMC11195545; doi:10.7717/peerj.17375)
Supplement: Supplemental Information 1 [file peerj-12-17375-s001.docx]

*Supplementary material*

Table S1: Pearson correlation between environmental variables. Temperature = average temperature, Water in soil = average water in soil, Productivity = average SAVI index, Mean annual temperature and Annual precipitation.

|  | Productivity | Temperature | Water in soil | Mean annual temperature | Annual precipitation |
| --- | --- | --- | --- | --- | --- |
| Productivity | 1.000 | 0.810 | -0.834 | 0.960 | 0.972 |
| Temperature | 0.810 | 1.000 | -0.918 | 0.873 | 0.883 |
| Water in soil | -0.834 | -0.918 | 1.000 | -0.911 | -0.937 |
| Mean annual temperature | 0.960 | 0.873 | -0.911 | 1.000 | 0.970 |
| Annual precipitation | 0.972 | 0.883 | -0.937 | 0.970 | 1.000 |


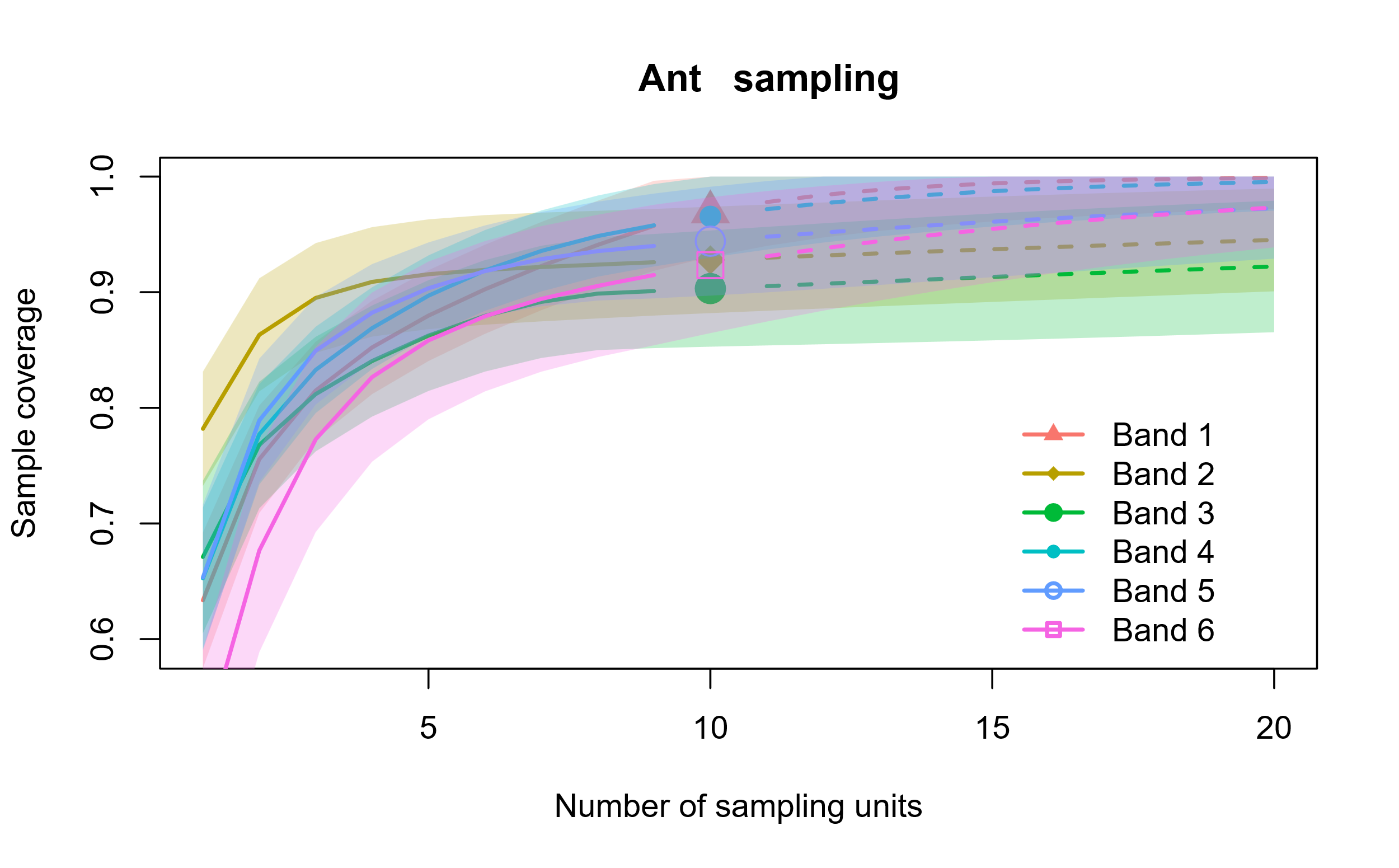

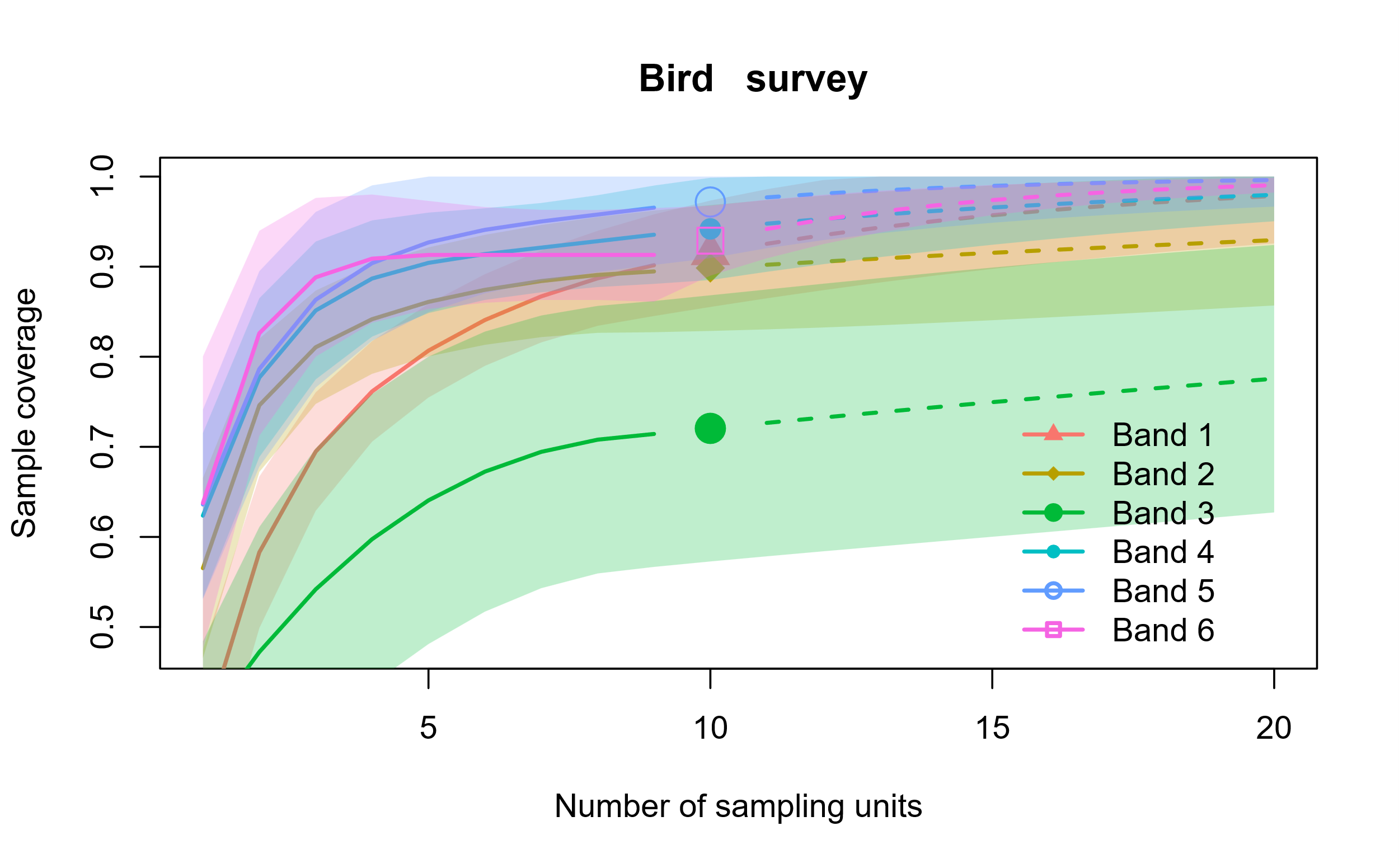

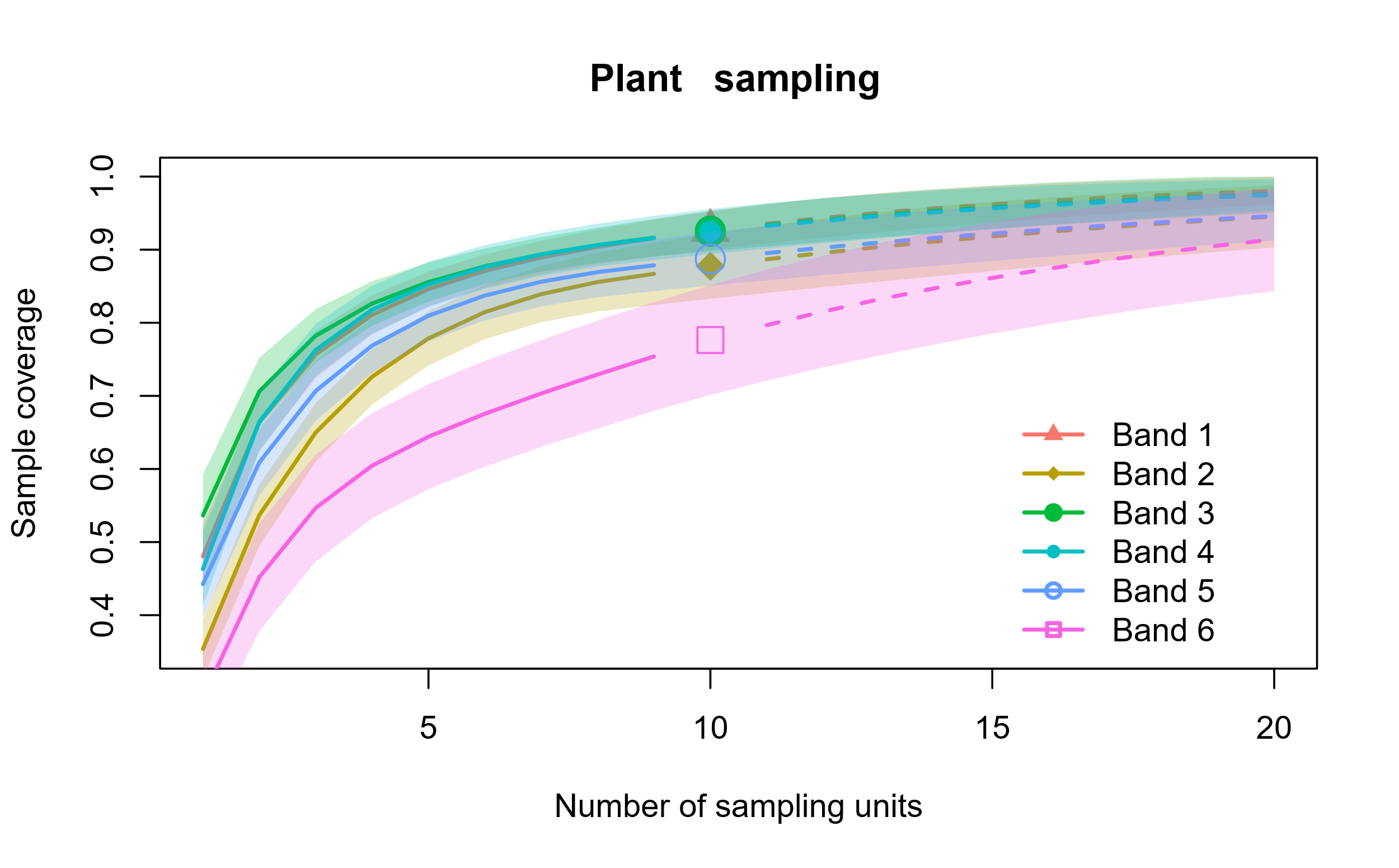

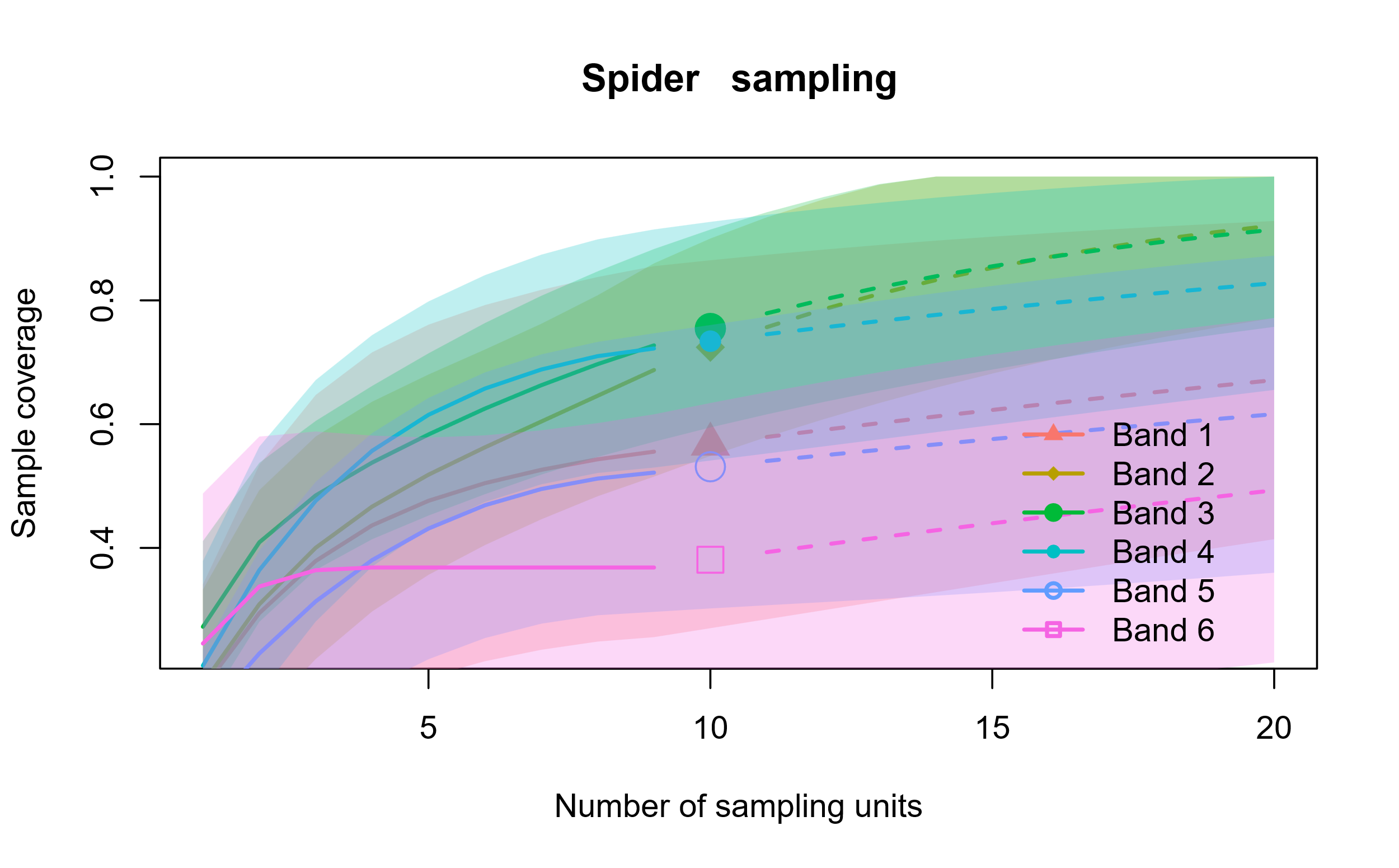


Figure S1: Sample coverage per taxon for each elevation band (from the base upwards). Proportion of species recorded as a function of the number of sampling units. The lines represent the expected coverage curve for a random community as the number of sampling units increases, with 95% confidence intervals. The dots above the lines indicate the number of sampling units (10).

Table S2: Variance inflation factor (VIF) for the models with values less than five. Temperature = average temperature, Water in soil = average water in soil, Productivity = average SAVI index, Mean annual temperature and Annual precipitation.

| Response variables | independent variable 1 | VIF | independent variable 2 | VIF |
| --- | --- | --- | --- | --- |
| Ant richness | Temperature | 2.54 | Productivity | 2.54 |
| Bird richness | Temperature | 2.33 | Productivity | 2.33 |
| Plant richness | Temperature | 3.67 | Mean annual temperature | 3.67 |
| Ant nestedness | Temperature | 2.31 | Productivity | 2.31 |
| Spider turnover | Temperature | 2.57 | Productivity | 2.57 |
| Spider nestendess | Temperature | 2.19 | Productivity | 2.19 |
| Bird turnover | Water in soil | 2.98 | Productivity | 2.98 |
| Plant turnover | Temperature | 3.50 | Mean annual temperature | 3.50 |
| Plant nestedness | Temperature | 2.72 | Mean annual temperature | 2.72 |


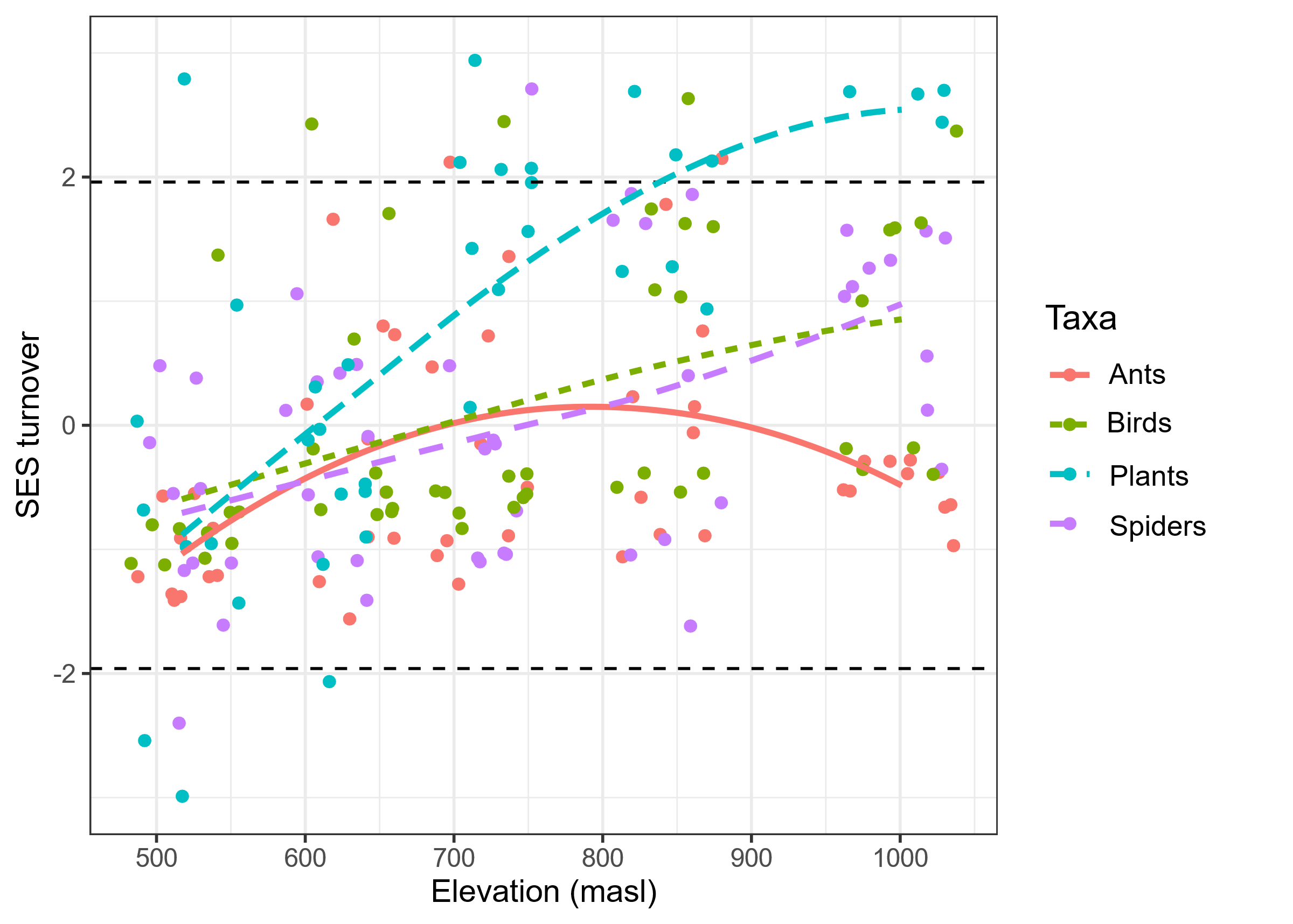

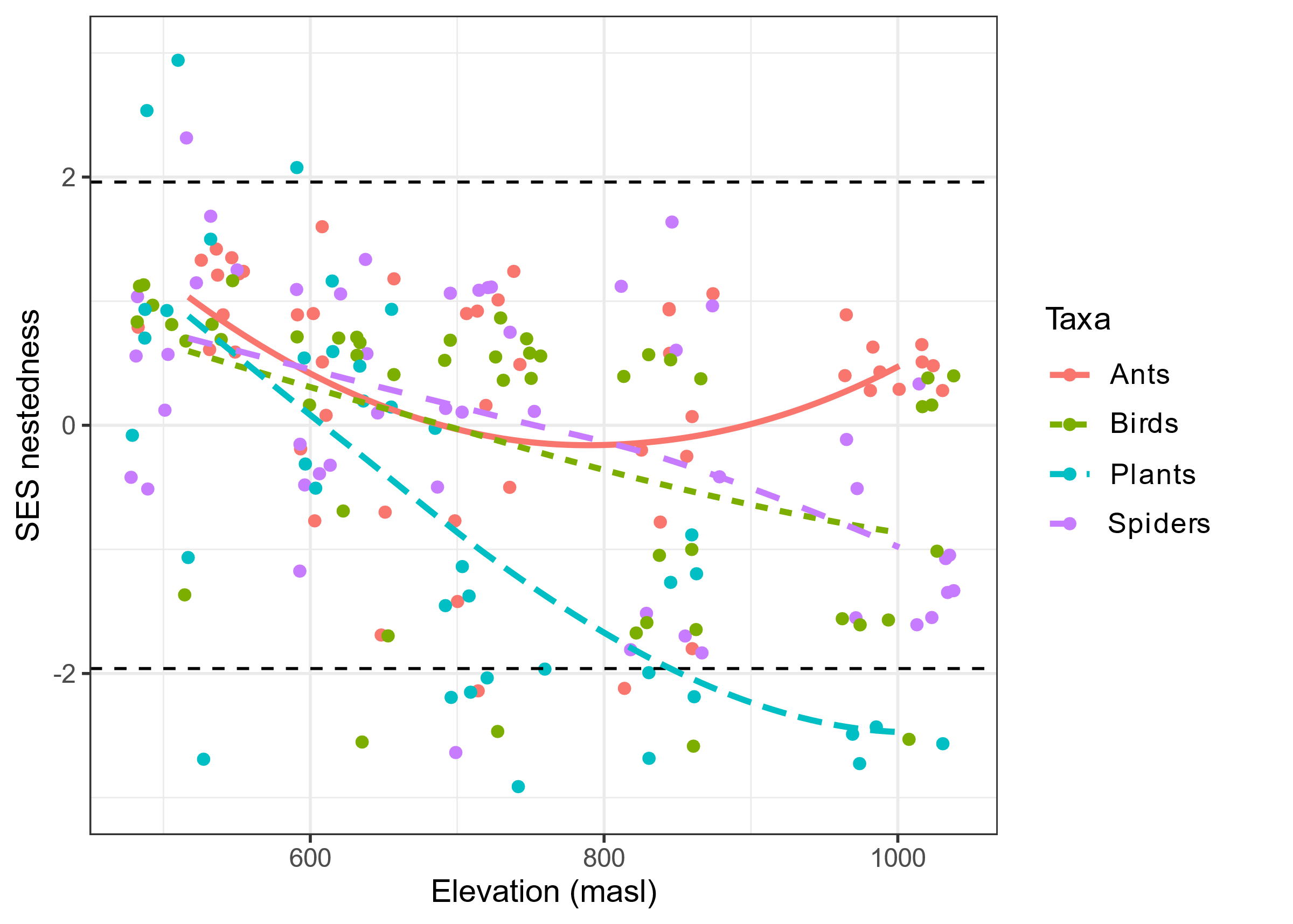

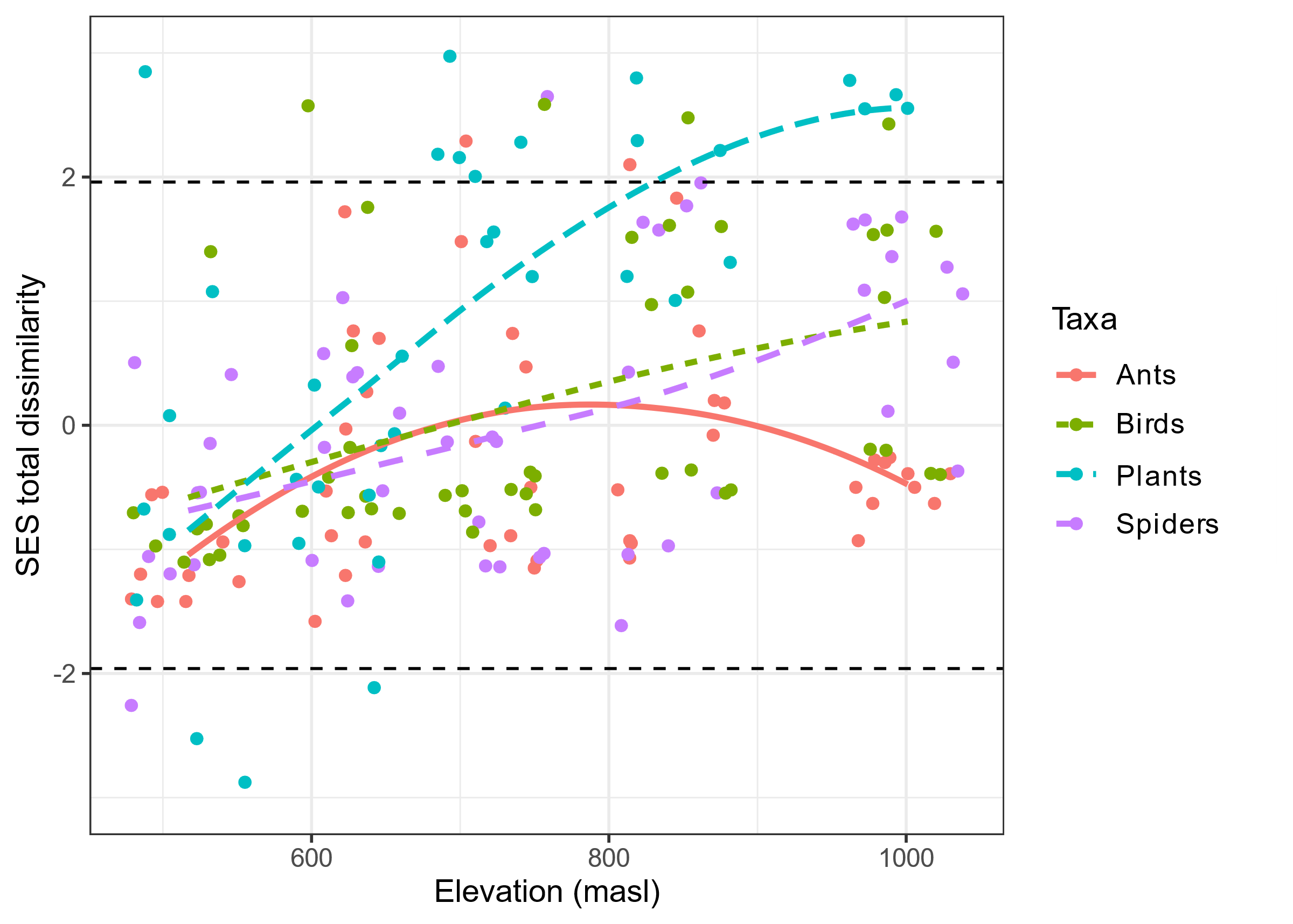


Figure S2: Standardized effect sizes (SES) of Taxonomic dissimilarity and its components between the lowest altitudinal band and each sampling site of the upper bands. Lines indicate the nonparametric method LOESS (span = 1.5) for each taxon. Dashed black lines indicate that values greater than 1.96 or less than -1.96 are significantly greater or less than expected (α = 0.05). The x-axis indicates the elevation (masl) of each upper band.

List of species collected or observed during fieldwork in the Ernesto Tornquist Provincial Park.

**Ants:**

*Acromyrmex* sp1

*Apterostigma pisolum*

*Brachymyrmex* sp1

*Brachymyrmex* sp2

*Camponotus fuscocinctus*

*Camponotus mus*

*Camponotus punctulatus*

*Camponotus rufipes*

*Camponotus* sp1

*Crematogaster* sp1

*Cyphomyrmex* sp1

*Dorymyrmex* sp1

*Eurhopalothrix bruchi*

*Gnamptogenys* sp1

*Hypoponera* sp1

*Linepithema micans*

*Monomorium* sp1

*Pheidole* sp1

*Pheidole* sp2

*Pheidole* sp3

*Pheidole* sp4

*Pheidole* sp5

*Pheidole* sp6

*Pogonomyrmex coarctatus*

*Pogonomyrmex naegelii*

*Solenopsis* sp1

*Solenopsis* sp2

*Solenopsis* sp3

*Solenopsis* sp4

*Solenopsis* sp5

*Mycetomoellerius* sp1

*Wasmannia williamsoni*

Spiders:

"*Lycosa*" *presumptuosa*

*Acanthogonatus centralis*

*Aillutticus nitens*

*Allocosinae* sp1

*Anyphaenidae* sp1

*Apopyllus pauper*

*Apopyllus silvestri*

*Arachosia* sp1

*Ariadna boesenbergi*

*Atomosphyrus breyeri*

*Camilina galianoae*

*Camilina pulchra*

*Camilina* sp1

*Caponina notabilis*

cf *Neomaso* sp1

cf *Sumampattus pantherinus*

cf *Tibellus* sp1

*Cybaeodamus ornatus*

*Dendryphantes* sp1

*Dictyna* sp1

*Drymusa serrana*

*Echemoides argentinus*

*Euryopis spinifera*

*Falconina gracilis*

*Goeldia* cf *luteipes*

*Goeldia* cf *patellaris*

*Goeldia* sp1

indet sp

indet sp1

indet sp2

*Latrodectus* sp1

*Lycosinae* sp1

*Meriola hyltonae*

*Misumenoides athleticus*

*Misumenops* sp1

*Monapia fierro*

*Odo bruchi*

*Pardosa birabeni*

*Petrichus* sp1

*Sanogasta* sp1

*Scytodes globula*

*Sphecozone* sp1

*Sphecozone* sp2

*Steatoda ancorata*

*Tmarus* sp1

*Tullgrenella lunata*

*Tullgrenella quadripunctata*

*Tullgrenella serrana*

**Birds:**

*Anthus correndera*

*Asthenes modesta*

*Athene cunicularia*

*Catamenia analis*

*Circus buffoni*

*Cistothorus platensis*

*Colaptes campestris*

*Embernagra platensis*

*Falco sparverius*

*Geositta cunicularia*

*Geranoaetus melanoleucus*

*Guira guira*

*Hymenops perspicillatus*

*Leistes loyca*

*Milvago chimango*

*Mimus saturninus*

*Molothrus bonariensis*

*Nothura maculosa*

*Pitangus sulphuratus*

*Progne elegans*

*Pseudoleistes virescens*

*Pyrocephalus rubinus*

*Sicalis flaveola*

*Sicalis luteola*

*Spinus magellanicus*

*Sporophila caerulescens*

*Troglodytes aedon*

*Tyrannus melancholicus*

*Tyrannus savana*

*Vanellus chilensis*

*Zonotrichia capensis*

**Plants:**

| **Vascular plant species** | **Category** |
| --- | --- |
| *(=Chascolytrum brizoides) Calotheca brizoides (Lam.) Desv.* | NATIVE |
| *(=Chascolytrum uniolae) Poidium uniolae (nees) Matthei* | NATIVE |
| *(=Galactia marginalis) Nanogalactia heterophylla (Gillies ex Hook. & Arn.) L.P. Queiroz* | NATIVE |
| *(=Polygala linoides) Senega linoides* | NATIVE |
| *Abutilon terminale* | NATIVE |
| *Achyrocline satureioides* | NATIVE |
| *Acmella decumbens* | NATIVE |
| *Adesmia incana* | NATIVE |
| *Adesmia muricata* | NATIVE |
| aff. *Adesmia* sp. | NATIVE |
| aff. *Argyrochosma nivea* | NATIVE |
| aff. *Bromus bonariensis* | POSSIBLE ENDEMIC |
| aff. *Jarava juncoides* | NATIVE |
| aff. *Lucilia acutifolia* | NATIVE |
| aff. *Scolymus hispanicus* | ADVENTIVE |
| *Aira caryophyllea* | ADVENTIVE |
| *Anemone decapetala* | NATIVE |
| *Arenaria serpyllifolia* | ADVENTIVE |
| *Aristida murina* | NATIVE |
| *Aristida spegazzinii* | NATIVE |
| *Arjona tuberosa* | NATIVE |
| *Asteraceae* sp. |  |
| *Baccharis rufescens* var. ventanicola | ENDEMIC |
| *Baccharis ulicina* | NATIVE |
| *Bipinnula penicillata* | NATIVE |
| *Bromus catharticus* | NATIVE |
| *Bromus* sp. | NATIVE |
| *Cactaceae* sp. | NATIVE |
| *Cardionema ramosissima* | NATIVE |
| *Carex distenta* | NATIVE |
| *Carex phalaroides* | NATIVE |
| *Cerastium arvense* | ADVENTIVE |
| *Chaptalia integerrima* | NATIVE |
| *Chaptalia piloselloides* | NATIVE |
| *Chaptalia* sp. | NATIVE |
| *Chascolytrum subaristatum* | NATIVE |
| *Cheilanthes buchtienii* | NATIVE |
| *Chevreulia sarmentosa* | NATIVE |
| *Cinnagrostis viridiflavescens* var. *montevidensis* | NATIVE |
| *Cliococca selaginoides* | NATIVE |
| *Convolvulus hermanniae* | NATIVE |
| *Convolvulus laciniatus* | NATIVE |
| *Conyza monorchis* | NATIVE |
| *Crassula connata* | NATIVE |
| *Crepis setosa* | NATIVE |
| *Crocanthemum brasiliense* | NATIVE |
| *Cuphea glutinosa* | NATIVE |
| *Danthonia cirrata* | NATIVE |
| *Daucus pusillus* | NATIVE |
| *Dichondra sericea* var. *holosericea* | NATIVE |
| *Dichondra sericea* var. *sericea* | NATIVE |
| *Echium plantagineum* | ADVENTIVE |
| *Eleusine tristachya* | NATIVE |
| *Elionurus muticus* | NATIVE |
| *Eragrostis lugens* | NATIVE |
| *Eryngium* aff. *horridum* | NATIVE |
| *Eryngium* aff. *paniculatum* | NATIVE |
| *Eryngium horridum* | NATIVE |
| *Eryngium nudicaule* | NATIVE |
| *Eryngium paniculatum* | NATIVE |
| *Eryngium stenophyllum* | NATIVE |
| *Euphorbia* aff. *spathulata* | ADVENTIVE |
| *Evolvulus sericeus* var. sericeus | NATIVE |
| *Facelis retusa* | NATIVE |
| *Festuca australis* | NATIVE |
| *Festuca megalura* | INTRODUCED |
| *Festuca ventanicola* | ENDEMIC |
| *Galium richardianum* | NATIVE |
| *Gamochaeta americana* | NATIVE |
| *Gamochaeta filaginea* | NATIVE |
| *Geranium albicans* | NATIVE |
| *Glandularia peruviana* | NATIVE |
| *Glandularia platensis* | NATIVE |
| *Glandularia tenera* | NATIVE |
| *Gomphrena elegans* | NATIVE |
| *Gomphrena perennis* | NATIVE |
| *Grindelia ventanensis* | ENDEMIC |
| *Gymnocalycium reductum* var. *platense* | NATIVE |
| *Gyptis tanacetifolia* | NATIVE |
| *Helenium uniflorum* | NATIVE |
| *Helosciadium nodiflorum* | ADVENTIVE |
| *Herbertia lahue* | NATIVE |
| *Hieracium chacoense* | ENDEMIC |
| *Holocheilus brasiliensis* | NATIVE |
| *Hordeum* aff. *euclaston* | NATIVE |
| *Hypochaeris neopinnatifida* | NATIVE |
| *Hypochaeris pampasica* | NATIVE |
| *Hypochaeris petiolaris* | NATIVE |
| *Hypochaeris radicata* | ADVENTIVE |
| *Hypochaeris variegata* | NATIVE |
| *Jarava plumosa* | NATIVE |
| *Juncus* aff. *capillaceus* | NATIVE |
| *Juncus* aff. *microcephalus* | NATIVE |
| *Juncus imbricatus* | NATIVE |
| *Juncus* sp. |  |
| *Juncus uruguensis* | NATIVE |
| *Koeleria kurtzii* | NATIVE |
| *Krapovickasia flavescens* | NATIVE |
| *Lathyrus subulatus* | NATIVE |
| *Lathyrus tomentosus* | NATIVE |
| *Lathyrus tomentosus* | NATIVE |
| *Lepidium tandilense* | NATIVE |
| *Lucilia acutifolia* | NATIVE |
| *Luzula excelsa* | NATIVE |
| *Lysimachia verna* | NATIVE |
| *Mandevilla petraea* | NATIVE |
| *Margyricarpus pinnatus* | NATIVE |
| *Melica* aff. *rigida* | NATIVE |
| *Melica rigida* | NATIVE |
| *Microgyne trifurcata* | NATIVE |
| *Mimosa rocae* | NATIVE |
| *Nassella* aff. *filiculmis* | NATIVE |
| *Nassella megapotamia* | NATIVE |
| *Nassella melanosperma* | NATIVE |
| *Nassella* sp. | NATIVE |
| *Nassella trichotoma* | NATIVE |
| *Neja pinifolia* | NATIVE |
| *Neja pinifolia* | NATIVE |
| *Nothoscordum bonariense* | NATIVE |
| *Oenothera bahia-blancae* | NATIVE |
| *Oenothera indecora* | NATIVE |
| *Oenothera odorata* | NATIVE |
| *Oenothera parodiana* | NATIVE |
| *Oxalis articulata* | NATIVE |
| *Oxalis conorrhiza* | NATIVE |
| *Panicum* aff. *bergii* | NATIVE |
| *Panicum bergii* | NATIVE |
| *Parodia submammulosa* | NATIVE |
| *Paronychia brasiliana* | NATIVE |
| *Paspalum plicatulum* | NATIVE |
| *Paspalum quadrifarium* | NATIVE |
| *Pavonia cymbalaria* | NATIVE |
| *Petrorhagia nanteuilii* | ADVENTIVE |
| *Pfaffia gnaphaloides* | NATIVE |
| *Piptochaetium hackelii* | NATIVE |
| *Piptochaetium lasianthum* | NATIVE |
| *Piptochaetium lejopodum* | NATIVE |
| *Piptochaetium medium* | NATIVE |
| *Piptochaetium montevidense* | NATIVE |
| *Piptochaetium napostaense* | NATIVE |
| *Piptochaetium stipoides* var. stipoides | NATIVE |
| *Plantago myosuros* | NATIVE |
| *Plantago ventanensis* | ENDEMIC |
| *Pleopeltis pinnatifida* | NATIVE |
| *Poa* aff. *liguraris* | NATIVE |
| *Poa* sp. |  |
| *Podocoma hieracifolia* | NATIVE |
| *Polycarpon tetraphyllum* | ADVENTIVE |
| *Polystichum plicatum* | NATIVE |
| *Pombalia parviflora* | NATIVE |
| *Pseudognaphalium cheiranthifolium* | NATIVE |
| *Rhynchosia diversifolia* | NATIVE |
| *Rubus ulmifolius* | ADVENTIVE |
| *Schizachyrium spicatum* | NATIVE |
| *Scleranthus annuus* | ADVENTIVE |
| *Senecio pulcher* | NATIVE |
| *Senecio ventaniensis* | ENDEMIC |
| *Silene argentina* | NATIVE |
| *Sisyrinchium chilense* | NATIVE |
| *Sisyrinchium minutiflorum* | NATIVE |
| *Sisyrinchium pachyrhizum* | NATIVE |
| *Sisyrinchium platense* | NATIVE |
| *Sommerfeltia spinulosa* | ENDEMIC |
| *Sorghastrum pellitum* | NATIVE |
| *Stevia satureiifolia* | NATIVE |
| *Tagetes minuta* | NATIVE |
| *Thelesperma megapotamicum* | NATIVE |
| *Tragia geraniifolia* | NATIVE |
| *Tragia pinnata* | NATIVE |
| *Trifolium polymorphum* | NATIVE |
| *Turnera sidoides* L. pinnatifida Ssp. | NATIVE |
| *Vicia setifolia* var. bonariensis | NATIVE |
| *Viola arvensis* | ADVENTIVE |
| *Wahlenbergia linarioides* | NATIVE |
| *Wedelia buphtalmiflora* | ENDEMIC |
| *Woodsia montevidensis* | NATIVE |
